# Capability accumulation and product innovation: an agent-based perspective

**DOI:** 10.1007/s00191-021-00732-9

**Published:** 2021-06-24

**Authors:** Claudius Gräbner, Anna Hornykewycz

**Affiliations:** 1 Institute for the Comprehensive Analysis of the Economy (ICAE), Johannes Kepler University Linz, Linz, Austria; 2 Institute for Socioeconomics, University of Duisburg-Essen, Duisburg, Germany; 3 ZOE. Institute for Future-Fit Economies, Bonn, Germany

**Keywords:** Agent-based modeling, Capabilities, Complexity, Learning, Innovation, B52, C63, D21, L23, O33

## Abstract

This paper studiesthe relevance of productheterogeneity and relatedness for the accumulation ofcapabilities in firms, as well as their implications for innovation dynamics. The existing literature has produced extensive evidence on the relevance of capability accumulation for innovation processes. Yet, an assessment of prior attempts to model these processes indicates that when it comes to the final consumption good sector, the evolutionary macroeconomic literature has focused on process rather than product innovation. To facilitate the consideration of empirical and microeconomic insights on product innovation in these models, this paper introduces a simple agent-based model, which may later serve as an innovation module in macroeconomic models. In the model, firms accumulate capabilities to produce final consumption goods that are heterogeneous in terms of their complexity and differ in their relatedness to each other. The model is used to study theoretical implications of different topological structures underlying product relatedness by conducting simulations with different ‘product spaces’. The analysis suggests that the topological structure of the product space, the assumed relationship between product complexity and centrality, as well as the relevance of product complexity in price setting dynamics have significant but nontrivial implications and deserve further attention in evolutionary macroeconomics.

## Introduction

The accumulation of capabilities has been found to be an important driver both for the successful development of individual firms (e.g. Aharonson and Schilling^2016^) as well as for the development of national economies (e.g. Hidalgo and Hausmann 2009). The empirical literature on the accumulation of capabilities and its implications for product innovation is considerable and has identified a number of stylized facts, both on the firm and aggregate level (for a recent review see Aistleitner et al. 2021).

On the theoretical side, models on the microeconomic level have been exceptionally successful in formalizing the process of product innovation in a way that is consistent with many of these empirical findings: in these models, product innovation takes place in a path dependent fashion since new inventions are a creative recombination of existing ideas (e.g. Caiani^2017^), and depends, for instance, on the collaboration among firms (e.g. Savin and Egbetokun 2016), human capital accumulation, as well as the engagement in R&D activities (e.g. Pyka et al. 2018). The integration of empirical insights is more difficult for macroeconomic models: when one aims at a comprehensive representation of a complete economy, innovation is only one among many important processes. The consideration of empirical specificities in such models has necessarily been more selective and, at least when it comes to the consumption good sector, the focus of the evolutionary macroeconomic literature so far has been rather exclusively on process innovation.^1^ More precisely, the innovations of firms in the capital sector usually impact the firms in the consumption sector by increasing their productivity values with regard to existing products (see, e.g., Dosi et al. 2019a; Caiani et al.^2020^), not by providing them with the ability to produce new products of different degrees of complexity. Similarly, innovations that happen directly within the consumption good sector mostly take the form of investments into R&D, resulting, if successful, in an increase of the productivity of that firm (see, e.g., Dawid and Delli Gatti 2018; Aistleitner et al. 2021). Thus, product innovation in the consumption good sector has received relatively little attention, which leaves the topic of the creation of new consumption goods largely understudied (for one of the few exceptions so far see Ciarli et al. 2018). Given the greater challenge that comes with the modelling of product innovation, this current state is understandable. At the same time, it also represents an important avenue for further research: for theoretical reasons – because product innovation is an important element in evolutionary theorizing (e.g. Dopfer et al. 2004) – and for empirical reasons – since product invention is a crucial mechanism underlying economic development (see, e.g., Hausmann et al. 2007; Hidalgo et al. 2007; Hidalgo and Hausmann^2009^).

Against this backdrop, the present paper aspires to make two contributions to the existing literature: first, it is meant as a first step in bridging the – thus far complementary – results of micro- and macroeconomic models: it takes up ideas developed in the microeconomic context – such as the invention of new products on a technology space – and formulates them in a manner that is not only more consistent with how innovation processes are modelled in macroeconomic models, but also so simple that the model could be used as a module within a more comprehensive macroeconomic framework. This could allow for the exploration of interaction effects between the mechanisms which, so far, have only been studied separately on the micro- and macroeconomic level.

Second, and related to the first goal, the paper takes up important empirical results and explores their theoretical implications for innovation processes in a microeconomic setting. More precisely, focusing on product innovation, it studies how different structures of product relatedness affect the innovation activities of firms. In most of the existing literature, innovation processes do not follow a particular structure, yet the empirical literature has shown that products are related to each other in a systematic way, and that the structure of these relationships matters (Hidalgo et al. 2007). The present paper explores the theoretical implications of these findings.

To achieve these goals, the paper proceeds as follows: the next section substantiates the motivation of the paper and reviews the relevant literature. Section 3 introduces the model, the results of which are described in Section 4. A discussion of the results follows in Section 5. Section 6 summarizes the implications for future research and concludes the paper.

## Motivation and literature review

As indicated above, the overall goal of the present paper is to introduce a model of capability accumulation that, first, takes ideas from the microeconomic literature on how product innovation takes place and formulates them in a framework that is more similar to how macroeconomic models are built; and that, second, explores the theoretical implications of recent empirical results on the relatedness of products and innovation processes. In this section, we justify such an endeavour and align the contribution with the existing literature.

### Motivation: the case for models bridging the micro and macro ABM literature

Why is the development of models that bridge the micro and macro literature in the context of capability accumulation promising, and how does such an approach differ from what evolutionary macroeconomic agent-based models currently do? With regard to the relevance of capability accumulation, there is now a broad consensus that they are empirically relevant on both the micro and macro level: in the macroeconomic literature it is argued that “[...] countries tend to approach the level of income associated with the capability set available in them” (Hidalgo and Hausmann 2009, p. 10570), and on the micro level a “firm’s technological capabilities are central to its identity, its strategies, and its potential for success” (Aharonson and Schilling 2016, p. 81). Statements such as these are underpinned by a vast amount of empirical findings both for the macro- as well as the firm-level (for a review see Aistleitner et al. 2021) and, thus, are calling for models that integrate both micro- and macroeconomic dynamics. Moreover, such joint consideration of the different levels is a constituent element of evolutionary (e.g. Dopfer et al.^2004^) and systemist (e.g. Gräbner and Kapeller 2015, 2017) research in general and, thereby, also desirable from a more theoretical perspective as well.

This integrated analysis of the micro and the macro level is exactly what macroeconomic agent-based models have been developed for (Hanappi and Scholz-Wäckerle 2017; Dosi and Roventini 2019). Such models, which aim for a comprehensive representation of the overall economy, however, face trade-offs when it comes to portraying complex mechanisms such as capability accumulation: by their nature they must consider all aspects of an economy including households, firms, private and central banks as well as governments. Understandably, not all of them can be represented in maximum detail. Such an approach would go against the central idea of models, i.e. to focus on the essential aspects of the system under investigation. Moreover, it would also be practically infeasible.^2^ Thus, even when agent-based models are meant to integrate mechanisms on the micro *and* macro level, they tend to take a bird’s-eye view on what happens on the micro level, and focus on those aspects for which established routines and guideline models from the micro do exist. Thus, there is room for models that bridge the gap between micro and macro and to prepare the integration of certain mechanisms into macro models at a later stage. But not only do we hope that the model introduced below successfully synthesizes work from evolutionary microeconomics and can later serve as a module within macroeconomic ABM – such intermediate models can also facilitate a feedback from macroeconomic models back into microeconomics: Firstly, there are a number of mechanisms that affect capability accumulation that so far have been considered *only* in the macroeconomic ABM literature, such as the role of industrial policy or public research.^3^ Secondly, some insights, such as the network of product relatedness (Hidalgo et al. 2007), originally come from the field of macroeconomics and, finally, mechanisms studied in microeconomics in isolation might function differently when they operate within a broader macro-like framework. To explore this possibility, models that include mechanisms from both the micro and the macro literature but do not aim for a fully comprehensive representation of the overall economy are necessary.

### Previous research I: capability accumulation in macroeconomic ABM

Processes of capability accumulation are considered in the macroeconomic ABM literature mainly under the topics of ‘innovation’ and/or ‘technological change’. Although the various model families differ in details, a number of standard ways to model innovation have emerged. All of them stress the relevance of R&D investments, and most of them – at least when it comes to consumption goods – focus on process innovation, i.e. the accumulation of productive capabilities that make firms more productive, rather than enabling them to produce more or different products – with the exception of Ciarli et al. (2018), which we will elaborate on below.

While most models feature both a consumption and a capital good sector, the locus of capability accumulation differs: In Dosi et al. (2019b) and Caiani et al. (2019) capability accumulation takes place in the consumption good sector. Investment into R&D increases the chances of firms to *innovate* – i.e. to increase their labour productivity – or to *imitate* – i.e. to copy the production technology of other firms, which might also result in increased labour productivity. Rengs et al. (2020) extend upon the same logic by adding ecological concerns to the firms’ decision problem: firms invest into R&D and then decide whether they try to increase their labour productivity or reduce CO_2_ emissions associated with the production of their consumption goods. Here, the effect of the investment does not only depend on the firm itself, but also on spillovers from firms in its environment.

Ciarli et al. (2018) consider capability accumulation in both the consumption and capital good sector: in the latter, investments into R&D enable capital good firms to produce capital goods that increase the productivity of consumption good firms – and that are, therefore, easier to sell at higher prices (this mechanism is also used in Hötte 2021). In the consumption good sector, Ciarli et al. (2018) also feature (as one of few macro ABM) some kind of product innovation: when consumption good firms invest into R&D, they might come up with higher quality goods, which can then be sold to consumers at higher prices.^4^

A different kind of capability accumulation is discussed in Hötte (2021): in her model, employees learn to use certain capital goods and become more productive over time (‘learning-by-doing’). They can then take the tacit knowledge that was gathered in this way with them to other firms when they change their employer, thereby also adding a spillover dimension to the model. In the end, however, this process also leads to increased productivity. Consequently, it is in effect similar to the models discussed above.

This cursory review of the literature^5^ indicates that investment into R&D activities that improve productivity is by far the most common way to consider capability accumulation in macroeconomic ABM (see also Table 1). Beyond that, various indirect channels of capability accumulation can be found in these models, e.g. the effect of innovation policy, public research or different labour market institutions. Nevertheless, all of them ultimately affect capability accumulation via their effect on the R&D investment of firms. Since R&D investment is indeed one of the major determinants of innovation and capability accumulation, this is not a bad thing *per se*. Yet, it is important to keep in mind that such a treatment leaves aside the consideration ofproduct innovation in the consumption good sector – which certainly is most relevant for the developmental implications of innovation processes (Hausmann et al. 2007; Hidalgo et al. 2007). In alternative modelling frameworks, such as endogenous growth theory, the so-called *expanding variety models* that stand in the tradition of Grossman and Helpman (1991a) and Grossman and Helpman (1991b) *do* feature some kind of product innovation, yet even here, the focus is on product varieties rather than new products.^6^ Models that explain how firms learn to produce different products, such as those falling into different SITC or HC categories, are still to be developed on the macroeconomic level. The model discussed in Section 3 is intended to be a first step in that direction.

**Table 1:** Capability accumulation in macroeconomic ABM

Central mechanism	Examples
R&D investment	Ciarli et al. (2018), Dawid et al. (2019), Hötte (2021), Dosi et al. (2019b), Caiani et al. (2019), Rengs et al. (2020)
Spillovers among firms	Dosi et al. (2019b), Caiani et al. (2019), Rengs et al. (2020)
Worker’s experience	Dawid et al. (2019), Hötte (2021)

### Previous research II: capability accumulation in microeconomic ABM

A number of evolutionary models have been used to study processes of capability accumulation on the micro (and meso) level.

The approach of modelling distinct products as nodes on a technology space followed in Section 3 is similar to Caiani (2017): here, technological change corresponds to the firms exploring a network – the technology space – in which the nodes represent different technologies. Yet, the nodes of the technology space in Caiani (2017) represent technologies that allow firms to produce a homogeneous product more productively, not to produce different products. This is different in Wersching (2010), who uses a circular technology space where nodes actually represent different product varieties. In contrast to the model proposed in Section 3, he does not focus on *how* the structure of the knowledge space impacts innovation dynamics. Rather, he focuses on the distinction between *incremental* and *radical* innovation and their dynamics, the impact of differing degrees of competition among innovators, as well as the effect of different technological regimes such as ‘Schumpeter Mark I’ and ‘Mark II’. In Savin and Egbetokun (2016), firms are situated on a two-dimensional ‘knowledge space’ and need to distribute their R&D expenses between the creation of new knowledge and their absorptive capacities, the latter measuring their ability to absorb existing knowledge. Knowledge can spill over voluntarily if firms engage in research alliances, or involuntarily via absorptive capacities. The authors then go on to study the emerging alliance networks under various parameter constellations. Although their model is different with regard to the overall purpose, it is in this regard related to the one proposed below in Section 3. A more conceptual approach is taken by Silverberg and Verspagen (2005). In their model, new technologies build upon existing technologies on a percolation space. By doing so, the model captures the fundamental idea of relatedness between old and new technologies (or products) and is capable of reproducing several stylized facts, such as the size distribution of innovation. If compared with the models discussed before, this concept is more abstract and it is not suited to study the implications of different topological structures of product relatedness. However, it does allow for an open-ended technological evolution.

A slightly different perspective is described in Desmarchelier et al. (2018), where firms decide what products to produce and to export. Depending on their capabilities, they can move to products that are related to those that they currently produce. Values for relatedness are taken directly from empirical data, and firms take into account the number of competitors in their neighbouring products, as well as their complexity and expected prices. This way, the model is able to replicate the empirical observations of Hidalgo and Hausmann (2009) on the product space, i.e. the empirical network of products and their relatedness for numerous Asian countries. Such an approach is related to the one pursued here, yet it differs from the model below in that the mechanisms that are taken into account are modelled on a more aggregated level and it does not explore the implications of different product space topologies or complexity distributions.

An alternative to a technology space is to model information pieces directly, and to let firms recombine these information pieces into newer and more complex technologies. While this approach is missing two important advantages of the technology space – namely the simplicity when it comes to studying the implications of different topological structures of relatedness and the potential to be calibrated against data – it allows to model an open-ended technological progress.

One example for such an approach is Arthur and Polak (2006), who discuss a model in which the elements of technologies are logic circuits. Newer technological circuits are built from existing ones, and their performance is measured by letting the logical circuits perform some pre-defined logical tasks. The authors find that logical circuits become more and more complex and sophisticated over time. A similar idea is pursued in Vermeulen and Pyka (2014), where the authors also show how collaboration and information sharing among firms allows them to come up with more complex inventions. A more abstract approach is taken in the ‘Bit Economy’ as introduced by Angus and Newnham (2013), which is a highly idealized economy populated by a finite number of state automata that are processing existing, and developing new bit-strings. This process can be interpreted as developing new technologies or products. While the model functions without any structural assumptions on production or consumption, it nevertheless does replicate some stylized facts of innovation processes, such as the relatedness of patents and growth of innovations. Its design makes it, however, much more abstract than the one we introduce in Section 3 below. It is less concerned with the economic mechanisms underlying innovation processes and can be understood as a general thought experiment on how more complex technologies emerge over time.

A more applied model that explicitly represents the process of re-combining existing technologies into new ones is presented in Pyka et al. (2019), who focus on three major determinants of capability accumulation on the firm and regional level: *R&D investment*, *alliances* and *learning-by-doing*. New products are assembled by recombining existing knowledge units and can then be sold to consumers. Firms accumulate capabilities by acquiring new knowledge units. This might happen (a) via direct investment into R&D, which creates new knowledge units that are necessarily similar to existing ones, (b) by copying those parts of knowledge units from partner firms that are not tacit and (c) simply via *learning by doing*. The main focus of this is not on the exploration of new technologies by the firms but rather on the investigation of channels of firm interaction and cooperation, as well as of the effectiveness of policies fostering cooperation.

In all, as indicated in Table 2, microeconomic models tend to concentrate on a different set of mechanisms than the macroeconomic models discussed in the previous section. Here, the focus is less on mechanisms involving the state and institutions, but more on mechanisms operating within or between single firms. As the next section shows, both literature branches, thereby, point to aspects of capability accumulation that were highlighted in the empirical research so far.

**Table 2:** Capability accumulation in microeconomic ABM

Central mechanisms	Examples using a technology space	Examples using an explicit representation of knowledge units
Alliances and cooperation	Savin and Egbetokun (2016)	Tur and Azagra-Caro (2018), Pyka et al. (2019)
Absorptive capacities & spillovers	Wersching (2010), Caiani (2017)	Pyka et al. (2019)
Recombination of existing knowledge	Silverberg and Verspagen (2005)	Arthur and Polak (2006), Angus and Newnham (2013), Vermeulen and Pyka (2014)

### Previous research III: the empirics of capability accumulation and product innovation

The empirical literature on capability accumulation is large and distributed among various disciplines (for a recent review see Aistleitner et al. 2021). Table 3 lists some exemplary references for the empirical results that are relevant for the design of the model introduced below.

**Table 3:** Empirical results on the determinants of firm capabilities

Relevant factor/result	Selected references
Absorptive capacitites	Chuang and Hobday (2013), Chung and Lee (2015), and Figueiredo and Cohen (2019)
Alliances & spillovers	Cantwell and Zhang (2013), Wu and Wei (2013), and Subramanian et al. (2018)
Experience and *learning-by-doing*	Villar et al. (2012) and Dosi et al. (2020)
Firm governance structure	Figueiredo (2008) and Collinson and Wang (2012)
Labour market institutions	Kleinknecht et al. (2016) and Cetrulo et al. (2019)
R&D spending	Figueiredo (2008), Wu and Wei (2013), and Chung and Lee (2015)
Relatedness of innovations	Neffke and Henning (2013), Aeron and Jain (2015), and Hidalgo et al. (2018)

One of the most frequently highlighted factors that determine the accumulation of capabilities are *absorptive capacities* of firms, which have originally been defined as a firm’s ability “to recognize the value of new, external information, assimilate it, and apply it to commercial ends” (Cohen and Levinthal 1990, p. 128). While nowadays the definition of absorptive capacities varies throughout studies, there are two effects that authors largely agree upon: First, absorptive capacities make it easier for a firm to evaluate its environment and make it more adaptable to changes. Second, absorptive capacities make it easier to acquire *involuntary* spillovers, i.e. spillovers that occur without the cooperation of other firms.

Another empirical regularity that enjoys wide support is the idea of *relatedness*, i.e. the fact that new capabilities are somehow related to capabilities that already exist. For example, Neffke and Henning (2013) find that firms are more likely to diversify into industries that require skills similar to those that they already use and work with. Aeron and Jain (2015) gather evidence on how firms actually develop new insights by experimenting with recombinations of existing knowledge (‘bricolage’).

Not surprisingly, the empirical literature has also documented the relevance of R&D spending (e.g. Chung and Lee 2015), research cooperation (e.g. Subramanian et al. 2018) and learning-by-doing (e.g. Dosi et al. 2020). When it comes to the institutional environment, results are more nuanced. With regard to intellectual property rights, for instance, some find an overall positive impact on capability accumulation (e.g. Ang 2011), while others find the relationship to be inverted U-shaped (e.g. Donoso 2014). Others, again, question the positive effect of intellectual property rights altogether (e.g. Dosi et al. 2006). Similarly, the majority of studies on governance structures are mainly case studies that highlight the nuanced and diverse effects of different firm government structures (e.g. Figueiredo 2008; Collinson and Wang 2012). The situation is somewhat different when it comes to the role of labour market institutions. Here, it is found that regulations that foster more flexible labour markets tend to be detrimental for capability accumulation (e.g. Kleinknecht et al.^2016^; Cetrulo et al. 2019; Hoxha and Kleinknecht 2020). Thus, while it is clear that while the role of the institutional environment is an important area for further research, up till now, there are only few general results that present themselves immediately as candidate mechanisms for concrete models.

The model discussed below in Section 3 is not intended to be a comprehensive framework that accounts for all the stylized facts referenced in Table 3. Rather, the focus will be on a selection of stylized facts that are (1) uncontroversial and (2) have not yet gained a lot of attention in the literature. More precisely, the goal is to introduce a fully supply-determined model that features different final products, and that allows for the exploration of various topological structures of relatedness among them for innovation dynamics and their implications. This is an endeavour worth undertaking since the relatedness of products has been highlighted as an essential feature in the empirical literature, but has not been adequately considered in existing macroeconomic models. At the same time, while a number of microeconomic models do consider relatedness of innovations, there is no model that studies the implications of different topological structures, or the distribution of product complexity within the network. What is more, the model also considers the respective roles of absorptive capacities and R&D investment – two factors that have also been highlighted in the empirical literature.

To keep the complexity of the model manageable, it neither considers research cooperation among firms nor learning by doing. These channels have been at the centre of respectable models for quite a while anyway. Moreover, the study of different firm government structures is left for further research since the empirical literature has not yet come up with concrete and decisive results that could inform general models. These decisions also pay tribute to the goal of developing a model that is simple enough to be used as a module within macroeconomic models in the future.

## Model description

The model integrates a heterogeneous product space into an agent-based model of innovation. While it might ultimately be used as a bridging vehicle between micro- and macroeconomic models, the focus during this first step is exclusively on the production side of the economy. Therefore, the model does not feature a household sector and leaves the demand side largely unexplored. In effect, it abstracts from questions of stock-flow consistency. It does, however, feature the infrastructure that is suited to account for financial flows between the firm and a financial sector: Firms hold accounts at potentially heterogeneous banks and feature a proper balance sheet that tracks their assets and liabilities. Since the focus of this paper, however, is not on financial dynamics, the present version of the model does not leverage this functionality: firms do not finance their expenses via loans and do not apply for any credit. This reduces the role of the banking sector to a minimum: the sole purpose of the banks – for now – is to host all firm accounts with their respective deposits. The main priority, for the moment, is the investigation of the effects of different structural properties of the product space, yet it is worth pointing out that the infrastructure to include a financial sector already exists.

The model features *M* firms that produce *N* heterogeneous products. An overview over all symbols used throughout the paper is given in Table 4. The parameters of the model and the chosen baseline values are given in Table 6 below. Each product corresponds to a vertex on the product space, which is used to represent the relatedness between products and is explained more precisely below in Section 3.1. Firms are located on a specific vertex in the product space and produce the product that corresponds to their current location. Because of the focus on the supply side and firms’ innovation processes, the produced products are sold in markets with an exogenously given level of maximum real demand that serves as a constraint for the firms’ production planning. In their aim to gain higher profits, firms search the product space for more lucrative products in each time period (their particular search strategy will be explained more precisely below in Section 3.1). If a firm finds a more lucrative product, it invests into various kinds of capability enhancing measures in order to learn how to produce this product and – ultimately – to be able to adapt its production (i.e. to make a move on the product space). The main question pursued here is how different topological structures of the product space and different distributional assumptions on product complexity, as well as different capabilities of the firms affect the innovation and production dynamics of the model. Other important determinants, such as the relevance of the demand side and mechanisms operating within the labour market are left for future applications (see also Section 5).

**Table 4:** An overview over all symbols used throughout the model description

Symbol	Description
*M*, *N*	Number of firms and products
$\mathcal {G}(V, E)$	The product space network with vertices *V* and edges *E*
${v_{i}^{c}}$	Complexity of product *i*
*ω*(*e*_*jk*_)	The weighted distance between two products on the product space
*F*_*i*_	Number of firms on the market for product *i*
*p*_*i*_	Price for good *i*
*α*	Impact of product complexity on price
*q*_*ij*_	Real output of firm *j* of good *i*
*A*	Capital productivity
*k*_*j*_	Capital stock of firm *j*
*s*_*ij*_	Market share of firm *j* in market for *i*
*q*^*max*^	Real demand constraint
*K*_*i*_	Aggregate capital stock in market for good *i*
*R*_*j*_	Revenues of firm *j*
*C*_*j*_	Costs of firm *j* for producing output *q*_*ij*_
π_*j*_	Profits of firm *j*
${m_{j}^{t}}$	Deposits of firm *j*
*r*^*d*^	Interest rate on deposits
*r*^*l*^	Interest rate on loans
*ℓ*_*j*_	Outstanding loans of firm *j*
*𝜃*	Payback rate for loans
*I*_*k*,*j*_	Investment in capital goods of firm *j*
*δ*	Depreciation rate
Υ_*j*_	Range of vision of firm *j*
Υ_*max*_	Maximum range of vision
*I*_Υ,*j*_	Investment into the enhancement of the range of vision of firm *j*
*p*_Υ_	Influence of amount invested into Υ_*j*_ on investment success
*I*_*ρ*,*j*_	R&D investment of firm *j*
*p*_*ρ*_	Influence of amount invested into R&D on investment success
*I*_*σ*,*j*_	Spillover investment of firm *j*
*p*_*σ*_	Influence of amount invested into spillovers on investment success
$\mathbb {P}_{X, j}^{t}$	Probability for a capability accumulation measure *X* to be successful
${\rho _{j}^{t}}$	The R&D level of firm *j* in *t*
${\sigma _{j}^{t}}$	The level of spillover capabilities of firm *j* in *t*
*ϕ*_*ρ*_,*ϕ*_*σ*_	The amount by which *ρ* and *σ* are increased after successful investment into the firm’s capabilities

Sub- or superscripts *i* always refer to a specific product, *j* to a specific firm, and *t* to a specific time step

### The product space

The model features heterogeneous products that differ in their complexity and their mutual relatedness. To represent products we use an artificial product space that follows the empirical work of Hidalgo and Hausmann (2009) and that corresponds to the ‘technology space’ in the models discussed in Section 2.3. A product space is a *weighted* network $\mathcal {G}(V, E)$ with $V(\mathcal {G})=\{v_{1}, ..., v_{n}\}$ vertices and $E(\mathcal {G})=\{e_{1}, ..., e_{m}\}\subseteq V\times V$ edges. Any edge *e*_*i*_ ∈ *E* connects two vertices such that *e*_*jk*_ = 〈*v*_*j*_,*v*_*k*_〉 with *v*_*j*_,*v*_*k*_ ∈ *V*. Each vertex *v*_*i*_ represents one product that is characterized by its *complexity*, ${v_{i}^{c}}$, which is a continuous measure for the sophisticatedness of the product. To introduce a measure of relatedness, the *weighted* distance between two vertices *ϕ*(*v*_*j*_,*v*_*k*_) = *ω*(〈*v*_*j*_,*v*_*k*_〉) = *ω*(*e*_*jk*_) represents their similarity in terms of the capabilities needed to produce them, i.e. $\omega (e_{jk})={|{v_{k}^{c}}-{v_{j}^{c}}|}$. For practical reasons, the normalised difference of their complexity values is used: starting from *v*_*k*_, the normalized distance to *v*_*j*_ is $\omega (e^{N}_{jk})=\frac {\omega (e_{jk})}{\omega (e^{*}_{k})}$ where $\omega (e^{*}_{k})$ represents the maximum weighted distance between *v*_*k*_ and all vertices that are connected to it. It is important to point out the difference between the *topological* distance between two products – i.e. the number of vertices on the shortest path between them, not accounting for the weights in *ω*(*e*_*jk*_) – and the *weighted* distance, which measures the relatedness of two products, but not the number of vertices in between them.

Firms can move on the product space, i.e. they can in principle change the product they produce.^7^ Following the ‘principle of relatedness’ (Hidalgo et al. 2018), however, firms cannot arbitrarily diversify into the production of any other product but can only diversify along the edges of the product space. Moreover, the transition to more related products (represented, as explained above, by smaller edge weights) is easier than the transition to less related products.

Empirical product spaces are derived from export data and deviate strongly from simple and complete networks. Rather, they feature complex core-periphery-like structures (e.g. Hidalgo et al. 2007). To explore the theoretical implications of these findings, the present model can be used to study the impact of different topological structures and distributions of product complexity on innovation dynamics. To this end, distinct artificial product spaces with pre-specified properties are created and the resulting model dynamics for such specifications are investigated. More precisely, of interest are (1) the impact of different network topologies, (2) the relationship between product complexity and centrality, as well as (3) the relevance of complexity for prices (see also Table 5).^8^

**Table 5:** The structural properties of the product space to be studied in the model. A complete list of model parameters is given below in Table 6

Topology of the product space		
Network	Parameters	Baseline values
Complete	–	–
Power-law cluster	Edges wired from new nodes *m* and probability to close triples to triangles *p*	*m* = 4,*p* = 0.7
Random (‘Erdös-Rényi’)	Probability that an edge exists *p*	*p* = 0.25
Regular network	Degree of every node *m*	*m* = 4
Ring	Number of wired neighbors *m*	*m* = 2
Scale-free (‘Barabási-Albert’)	Edges wired from new nodes *m*	*m* = 4
Allocation of complexity values		
Kind	Description	
Random allocation	Complexity values are distributed randomly among all products.	
Weighted degree	Complexity correlates strongly with vertex weight.	
Eigenvector centrality	Complexity correlates strongly with eigenvector centrality.	
Closeness centrality	Complexity correlates strongly with closeness centrality.	

**Fig. 1:**
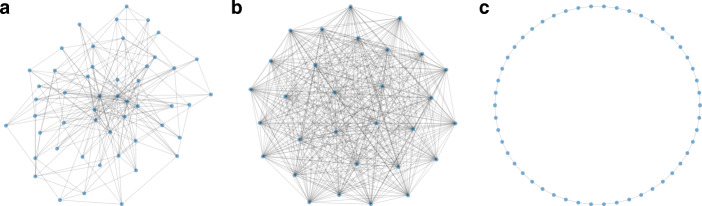
Different kinds of topologies for the product space. Panel **a** shows a scale-free (‘Barabási-Albert’), panel **b** a complete and **c** a ring network with 30 products each

With regard to different topologies, we distinguish between a *complete* network, in which each vertex is connected to every other vertex; a *regular* network, in which each vertex is connected to *m* other vertices; a *ring* where every vertex is connected to two neighbours; a *Barabási-Albert* network (Barabási and Albert 1999), which is characterized by its scale free degree distribution; a *power law cluster network* that is characterized by both a power law degree distribution and large clustering (Holme and Kim 2002), as well as a random (‘Erdös-Rényi’) network (Erdös and Rényi 1959) in which each edge exists with the same probability *p* (see Fig. 1 for an illustration).^9^

The model is also used to study whether the relationship between product complexity and centrality in the product space has an impact on the model dynamics. To this end, complexity values are either distributed randomly, or according to the weighted Degree, Eigenvector or Closeness centrality of the products.^10^ Finally, as will be described in more detail below in Section 3.3, the model is used to study how the influence of complexity on product prices and the firms’ ability to learn about their surroundings of the product space impacts the overall dynamics.

### Timeline of events

The model is analyzed via Monte Carlo simulations. For each parameter constellation, the model is run 50 times and the results are described via adequate summary statistics. Each single run begins with the allocation of the *M* firms, which are initially endowed with the same initial stock of capital and the same level of capabilities, on the product space. To ensure that firms start on different and peripheral locations of the product space, they are placed on the products with the lowest Eigenvector centrality. This also ensures that firms do not start on products with high complexity despite not having invested into the accumulation of their capabilities. Then, each of the *t* time steps proceeds as follows:

1. Prices of the produced products are computed
2. Firms produce output and realize profits
3. Firms choose a target product
4. Firms invest into their capital stock
5. Firms invest into various capability enhancing measures
6. If possible, firms move to their target product

The single steps are now described in more detail.

### Determination of prices

Under usual circumstances, prices in an ABM would form endogenously by firms offering products at prices, which depend on their production costs and their past selling experience, and consumers buying products on a consumption good market.^11^ However, since the present model is meant to focus on the production side of the economy and does not feature proper households, a more simplistic price function is assumed. When ${F_{i}^{t}}$ denotes the number of firms producing good *i* in period *t*, the price of good *i* is given by:

1 $$ {p_{i}^{t}} = \frac{{v_{i}^{c}} \cdot \alpha}{{F_{i}^{t}}}. $$

The parameter *α* is fixed and determines the impact of product complexity ${v_{i}^{c}}$ on the price of product *i*. Varying *α* across simulations allows for an exploration of how varying the relevance of product complexity for prices impacts the model dynamics (see Section 4 below). In principle, prices are assumed to be market clearing – however, as stated above, markets are constrained by some maximum real demand, an assumption that was made to ensure that firms could not endlessly accumulate profits, but that does not have important implications for the model result (see the sensitivity analysis in Section 4.4). Although the overall formula is too simplistic to count as a realistic representation of true price formation processes, it is sufficient for the purpose at hand since it captures both the fact that a larger and more competitive market comes, *ceteris paribus*, with lower prices and higher product complexity is, *ceteris paribus*, associated with higher prices. This assumption is consistent with the empirical results of Carlin et al. (2001) and Storm and Naastepad (2015), who show that the elasticity of demand to prices for more complex products is very low, but increases for less complex products.

### Production of goods and firm profits

Firms produce the product that corresponds to their current position on the product space using the capital stock they have accumulated so far. Output ${q}_{ij}^{t}$ of firm *j* of good *i* in *t* is given by

2 $$ \begin{array}{@{}rcl@{}} {q}_{ij}^{t}&=&\min \left[A \cdot k_{j}^{t-1}, s_{ij}^{t} \cdot q^{max}\right]\\ \text{with}\quad s_{ij}^{t} &=& \begin{cases} \frac{k_{j}^{t-1}}{K_{i}^{t-1}} & \text{ if the firm was already in the market}\\ \frac{k_{j}^{t-1}}{k_{j}^{t-1} + K_{i}^{t-1}} & \text{ if the firm is new in the market} \end{cases} \end{array} $$

where $k_{j}^{t-1}$ is the capital stock of firm *j* from the previous period and *A* is capital productivity. Since the focus is on *product* rather than *process* innovation, capital productivity is assumed to be constant ${A_{j}^{t}}=A \forall t, j$. Moreover, each market is constrained by an exogenously determined level of maximum real demand for each product, i.e. only a finite amount of $q_{i}^{max}$ can be sold for each product. If the market operates at this maximum output level, each firm produces output according to their market share $s_{ij}^{t}$, which is computed as their share of the total capital stock in the market $K_{i}^{t-1}$.^12^ It is worth noting that firms will not produce any output unless they know that revenues will exceed production costs, i.e. firms cannot make negative profits. These assumptions imply that firms have a lot of information on their current market. Since, however, we do not focus on the dynamics of a single market but rather on the transition of firms to different markets, it suffices for the problem at hand to assure that, *ceteris paribus*, more complex products and products associated with less competitive markets will lead to higher profits. In all, revenues ${R_{j}^{t}}$ and costs ${C_{j}^{t}}$ for firm *j* are computed as

3 $$ \begin{array}{@{}rcl@{}} {R_{j}^{t}} &=& {p_{i}^{t}} \cdot q_{ij}^{t} \end{array} $$

4 $$ \begin{array}{@{}rcl@{}} {C_{j}^{t}} &=& \mathcal{C} \cdot \frac{q_{ij}^{t} }{A} \end{array} $$

where general production costs are given as costs per unit of capital $\mathcal {C}$. Net revenues ${R_{j}^{t}} - {C_{j}^{t}}$ are then booked on the bank account of the firm, increasing its deposits ${m_{j}^{t}}$.

In a next step, firms would receive interests on their deposits, pay back a share of their loans and pay interests on their loans to the bank, leaving them with their final profit ${{\Pi }_{j}^{t}}$:

5 $$ {{\Pi}_{j}^{t}} = {R_{j}^{t}} - {C_{j}^{t}} + {r^{d}_{t}}\cdot m_{j}^{t-1} - {r^{l}_{t}}\cdot\ell_{j}^{t-1} - \theta \ell_{j}^{t-1}, $$

where ${r^{d}_{t}}$ and ${r^{l}_{t}}$ stand for the interest rates on deposits and loans, respectively, $\ell _{j}^{t-1}$ for the amount of outstanding loans of the firm before making its payback, and *𝜃* the payback rate for loans (thus, when the firm does not take up new loans we have ${\ell _{j}^{t}}=\left (1-\theta \right )\ell _{j}^{t-1}$). However, as indicated above, in the current scenario, firms do not receive any interest on their deposits and do not apply for any loans in order to finance their expenses. Hence, the parameters ${r^{d}_{t}}$, ${r^{l}_{t}}$ and *𝜃*, as well as *ℓ*_*j*_ are all equal to zero and the overall profits of firm *j*, ${{\Pi }_{j}^{t}}$, equals net revenues: ${{\Pi }_{j}^{t}}={R_{j}^{t}} - {C_{j}^{t}}$.

Since firms are in an ongoing search for better production options, they will invest some part of their profits into capability enhancing measures, i.e. measures that will help them learn how to produce other, more lucrative products. Before investment measures can be taken, however, firms need to check all known production options and choose a target product.

### Firms choose their target product

In each time step, firms search for information on more profitable production opportunities and – if they find one – they invest into different measures to increase their capabilities which may ultimately enable them to reach the more profitable product of their choice.^13^ Thus, before each firm decides on what capability enhancing measures to invest in, firms choose a target product that these measures will be aimed at.

First, firm *j* considers all products that are within its *range of vision* ${{\Upsilon }_{j}^{t}}$. The latter is represented by an integer number that corresponds to the number of products that the firm can *see* (i.e. knows about). Throughout each model run, firms have the chance to extend their range of vision, thereby broadening their information on the product space and enabling them to better assess their environment. The set of all visible products $\mathcal {V}_{j}^{t}=\{v_{1}, v_{2},...,v_{\Upsilon }\}$ consists of the ${{\Upsilon }_{j}^{t}}$ closest products (in the sense of ‘most related’) to the current position of the firm. Note that these are not necessarily immediate neighbour products: if the weighted distance to a product that is two vertices away is smaller than the weighted distance to an immediate neighbour, the latter might not be in $\mathcal {V}_{j}^{t}$, while the former will be. Initially, ${{\Upsilon }_{j}^{t}}$ is set to unity for all firms, indicating that each firm only knows about the closest product on the product space.

For each product in $\mathcal {V}_{j}^{t}$, the firm then computes the profit it expects if it were to actually produce that product. As a heuristic, the firm takes the current market size (i.e. the aggregated capital stock of all firms currently producing that product) to assess its potential market share based on the current period:

6 $$ \begin{array}{@{}rcl@{}} \hat{q}_{ij}^{t}&=&\min \left[A \cdot k_{j}^{t-1}, \hat{s}_{ij}^{t} \cdot q^{max}\right]\\ \text{where}\quad \hat{s}_{ij}^{t} &=& \frac{k_{j}^{t-1}}{k_{j}^{t-1} + K_{i}^{t-1}} \end{array} $$

The expected profit of firm *j* for product *i* is then given by:

7 $$ \begin{array}{@{}rcl@{}} \mathbb{E}_{i}\left( {{\Pi}_{j}^{t}}\right) = \hat{q}_{ij}^{t} p_{i}^{t-1} - \hat{C}_{j}^{t} \end{array} $$

where $\hat {C}_{j}^{t}$ are the costs the firm expects to incur for the production of $\hat {q}_{ij}^{t}$.

The ultimate target product, then, is the product for which the expected profit is highest. As already mentioned, the price of each product increases with its complexity and decreases with market size. Therefore, the chosen target product will not necessarily be the most complex product in the range of vision.

Since firms cannot move around the product space arbitrarily but can only move along one vertex at a time and their target product may lie several steps away, the desired target may not be in the firm’s immediate reach. In this case, which will be elaborated on below, it takes the firm more than one period to learn how to produce the target product and it can therefore, only change its production after preparing for the move for several periods.

### Investment decision and capability accumulation

In order to learn how to produce the target product, firms need some level of capabilities that corresponds to the complexity of their target. These capabilities can be accumulated by investing into three different capability enhancing measures. Two such mechanisms that have been highlighted in the existing literature are R&D activities and *absorptive capacities*. While the first comprises all measures that a firm can take to single-handedly learn how to produce another product, the latter comprises all measures that enhance a firm’s knowledge of its environment – which, simply put, enables voluntary as well as involuntary knowledge spillovers. In other words, R&D measures may help a firm to gather capabilities that it previously did not know about (although other firms might have) without any need for cooperation, while absorptive capacity measures help a firm to gather capabilities by studying their environment and learning from the spillovers that thereby occur.

In the present model, absorptive capacity measures are split into (1) measures to learn from other firms that produce some product and (2) measures to study the firm’s environment, i.e. to enhance their knowledge about the product space. In each period, if firms have chosen a target product different from their current product, they invest into all different kinds of capability enhancing measures. If the measure was successful, the firm’s according capability level will be increased (see below). That is, R&D investment will increase the firm’s R&D capabilities, investment into the investigation of the firm’s environment will increase the firm’s *range of vision* (and will, therefore, be referred to *I*_Υ_) and investment that is aimed at learning from other firms (labelled *spillover* investment, hereafter) will increase the firm’s capability to benefit from spillovers. The range of vision and the level of spillover capabilities are two aspects of the firm’s absorptive capacities, which, for technical as well as analytical reasons, are considered separately.

#### Investment decision

Firms are profit-seeking and their highest priority is to obtain as much profit as possible in their current market. To this end, they invest into the accumulation of their capital stock, thereby controlling their future output capacities. That is, firms cannot only invest into the accumulation of capabilities, but also into their capital stock. More precisely, firms aspire to be able to sell some desired output, $\hat {q}_{ij}^{t+1} = q_{ij}^{t} + q^{max} - {\sum }_{j=1}^{F} q_{ij}^{t} $, which is oriented on current output, but takes into account the option for expansion that is determined by the difference of the maximum real demand constraint and current production of all firms *F* in the market given by $q^{max} - {\sum }_{j=1}^{F} q_{ij}^{t}$. Investment in capital stock $I_{k, j}^{t}$ is then computed as:

8 $$ {I}_{k, j}^{t}= \begin{cases} \frac{\hat{q}_{ij}^{t+1}}{A} - (1 - \delta) \cdot k_{j}^{t-1} & \text{if } \hat{q}_{ij}^{t+1} \geq {q}_{ij}^{t}, \\ \delta \cdot k_{j}{t}    & \text{if the firm has a production target and } \hat{q}_{ij}^{t+1} < {q}_{ij}^{t}, \\ 0      & \text{otherwise} \end{cases} $$

where *δ* denotes the depreciation rate of capital. That is, if a firm does not intend to *extend* its production but is currently trying to *change* its production to a new target product, it intends to invest sufficiently in order to compensate for the depreciation of capital – thereby assuming that a firm that is currently planning a production change is not willing to decrease its production capacities. If, however, a firm neither intends to extend its production nor wishes to change its product (or even wishes to decrease its output) it will not compensate for capital depreciation.

Next, the firm decides on investments into capability enhancing measures. In general, a firm that is currently planning to change its production will invest both into spillovers and R&D as well as, additionally, into expanding its range of vision. However, since having information on its environment (i.e. the product space) is crucial for choosing successful production paths, firms will invest into their range of vision even if they do not currently plan to change their production. The amount that is invested into each capability accumulation measure depends on how investment increases the probability of success, i.e. the probability of actually enhancing capabilities. In all, investment into range of vision, ${I}_{\Upsilon , j}^{t}$, into spillovers, ${I}_{\sigma , j}^{t}$ and into R&D ${I}_{\rho , j}^{t}$ is determined as follows:

9 $$ \begin{array}{@{}rcl@{}} {I}_{\Upsilon, j}^{t} &=& {{\Pi}_{j}^{t}} \cdot \frac{p_{\Upsilon}}{p_{\Upsilon} + p_{\sigma} + p_{\rho}}, \end{array} $$

10 $$ \begin{array}{@{}rcl@{}} {I}_{\rho, j}^{t} &=& \begin{cases} {{\Pi}_{j}^{t}} \cdot \frac{p_{\rho}}{p_{\Upsilon} + p_{\sigma} + p_{\rho}} & \text{if the firm is planning to change production}\\ 0 & \text{otherwise} \end{cases} \end{array} $$

11 $$ \begin{array}{@{}rcl@{}} {I}_{\sigma, j}^{t} &=& \begin{cases} {{\Pi}_{j}^{t}} \cdot \frac{p_{\sigma}}{p_{\Upsilon} + p_{\sigma} + p_{\rho}} & \text{if the firm is planning to change production}\\ 0 & \text{otherwise} \end{cases} \end{array} $$

where *p*_Υ_, *p*_*σ*_ and *p*_*ρ*_ are parameters that determine the influence of investment into the range of vision, spillovers and R&D on the success of the capability accumulation measure.

Since we currently do not offer firms the possibility to apply for loans, firms’ actual investment will be constrained by their current profits and deposits. If, therefore, the entire demand for investment exceeds this constraint, actual investment in each category will be adjusted while keeping the share of each investment relative to total investment equal.

#### Capability accumulation

Whether the measures taken in order to enhance a firm’s capabilities were successful and actually lead to a higher level of corresponding capabilities is determined by a Bernoulli process, where probability $\mathbb {P}_{X}$ for capability enhancing measure *X* to successfully increase the firm’s capabilities is given by

12 $$  \mathbb{P}_{X, j}^{t} = \begin{cases} p_{X} - \frac{1}{I_{X, j}^{t}} & \text{if } I_{X, j}^{t} \geq \frac{1}{p_{X}} \\ 0 & \text{otherwise}. \end{cases} $$

If the range of vision ${{\Upsilon }_{j}^{t}}$ was successfully increased, the range of vision will be extended as:

13 $$ {\Upsilon}_{j}^{t+1} = {{\Upsilon}_{j}^{t}} + 1. $$

Note, however, that maximum range of vision is constrained by some maximum share of knowledge Υ_*max*_, i.e. there is a maximum amount of vertices that a firm can know of. The sensitivity of the results to changes in this parameter is explored below in Section 4.4.

Similarly, a success in R&D or in spillovers will, respectively, lead to:

14 $$ \begin{array}{@{}rcl@{}} \rho_{j}^{t+1} &=& {\rho_{j}^{t}} + \phi_{\rho} \end{array} $$

15 $$ \begin{array}{@{}rcl@{}} \sigma_{j}^{t+1} &=& {\sigma_{j}^{t}} + \phi_{\sigma}, \end{array} $$

where *ϕ*_*ρ*_ and *ϕ*_*σ*_ are parameters that determine the effect of successful capability accumulation.

### Firms make their move on the product space

In principle, a firm can move to another product in the product space if it has accumulated sufficient capabilities. Yet, following the principle of relatedness, firms cannot arbitrarily move around on the product space. Rather, whether a firm can reach its target product is decided in a two-step process.

First, if the target product is not a neighbour on the product space, it will take the firm several periods to prepare for the change of production. The number of periods necessary are given by the number of vertices that, taking the shortest path, lie between the firm’s current product and its target. Second, if the firm is allowed to move after preparing for several periods, it is assessed whether its capabilities suffice. This will be the case if (1) the firm’s level of R&D capabilities exceed the complexity of the target product or if (2) other firms are already in the market for the target product and spillover capabilities exceed the target’s complexity. After moving to a new product, the search for ever more lucrative production options will go on until the firm has found a position on the product space from which no better product is visible.

Finally, at the end of each period, the firm’s capital stock *k*_*j*_ and bank account *m*_*j*_ are updated:

16 $$ {k_{j}^{t}} = (1 - \delta) k_{j}^{t-1} + I_{k, ij}^{t} $$

and

17 $$ {m_{j}^{t}} = m_{j}^{t-1} + {{\Pi}_{j}^{t}} - I_{tot, j}^{t} , $$

where ${{\Pi }_{j}^{t}}$ are the firm’s profits and $I_{tot, j}^{t}$ are the firm’s total investment costs.

## Results

The model is analyzed using Monte Carlo simulations and is run 50 times with 500 time steps for each parameter constellation. The baseline parametrization is summarized in Table 6. In the following, the focus of the discussion will be on the impact of (1) different topological structures of the product space (Section 4.1), (2) the relationship between the product position within the product space and its complexity (Section 4.2) as well as (3) the overall impact of simultaneous changes in these dimensions (Section 4.3). In Section 4.4 a number of sensitivity tests are conducted. In case the reader wishes to replicate the results or conduct alternative simulation exercises, the code of the model is available on GitHub.^14^

**Table 6:** The baseline parametrization of the model

Parameter	Baseline value
Number of firms	100
Number of banks	1
Number of time steps	500
Number of products in the product space	100
Relevance of complexity *α*	100
Demand saturation *q*^*max*^	75
Initial capital *k*_0_	50
Depreciation rate	0.1
Productivity *A*	0.5
Cost share $\mathcal {C}$	0.25
Information success parameter *p*_Υ_	0.12
Spillover success parameter *p*_*σ*_	0.09
R&D success parameter *p*_*ρ*_	0.06
Effect of successful spillovers *ϕ*_*σ*_	0.5
Effect of successful R&D *ϕ*_*ρ*_	0.5
Initial range of vision Υ	1
Maximum share of knowledge *y*	0.85
Initial spillover capabilities *σ*	0.1
Initial R&D capabilities *ρ*	0.1

If not mentioned differently, the results in this section were derived using these parameter settings. The standard procedure to allocate complexity values to products is according to the products’ degree centrality (see Fig. 4)

### The impact of different product space topologies

**Fig. 2:**
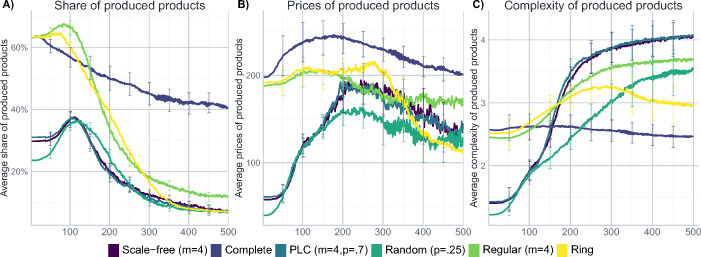
The dynamics of the model for different product space topologies. The remaining parameters are set as in Table 6. The lines show average results for 50 simulation runs, whiskers represent the standard deviation

**Fig. 3:**
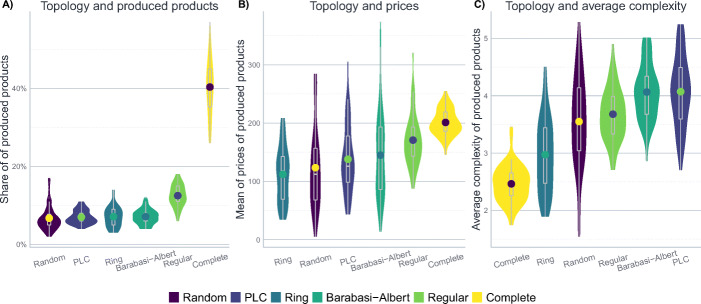
The effect of different product space topologies. The figure shows the distribution of results after 500 steps of 50 iterations of the model, i.e. the situation during the final time step in Fig. 2. The other parameters are set to their baseline level described in Table 6. The width of the violins represents the rotated kernel density of the results, the point within the violin corresponds to its mean. Boxplots represent the median and the inter-quartile range

Figure 2 represents the model dynamics for different topological structures of the product space. Noticeably, although the dynamics are not trivial, the state variables settle to a rather stable basin of attraction towards the end, even if not all variables approach a fixed point equilibrium. Therefore – and to get a better view on the inter-run variation – Fig. 3 visualizes the situation at the end of the simulation, i.e. at *t* = 500, using a violin- and a box-plot. This gives a more nuanced picture on the distribution of the results: the shape of the violin-plot is given by the rotated density of the distribution of results for the given outcome variable. From the additional box-plot within the violin, one can infer the median and the inter-quartile range of the results. For the sake of comparability with Fig. 2, the mean of the results is also represented with a dot within the violin plot. This kind of visualization maximizes the information about the final state of the simulation and allows for a better comparison of the parameter constellations.

The first immediate conclusion that can be inferred from Figs. 2 and 3 is that the topological structure of the product space does matter: there is apparent variation acrossparameter constellations, and the complete product space – which corresponds to the common implicit assumption that each product can be invented at any time – appears to be a particular special case. This is especially evident with regard to the share of produced products (Figs. 2a and 3a), where the variety of products develops similarly for all network topologies except for the complete network. That is, while for most topologies the amount of different products that actually get produced first rises and then declines (indicating a concentration of firms on only a few products after some diversification in the beginning), the results differ for the complete network where the concentration dynamics are much less distinct. For firms, it seems to be easier to reach the more complex center of the product space if the product space is less dense and fewer actual connections are available to them. In topologies that feature locally well-connected but globally rather isolated structures, firms tend to concentrate (or get stuck) in closely connected areas, which are more difficult to be left in favour of a different community of products, which might prevent firms from diversifying further. The implication of this is also visible once we consider the prices and complexity (Fig. 3b and c) of the produced products, where the complete network again takes a quite distinctive place in the results.

The particularity of the complete network is due to the fact that when all products are connected with each other, it is more difficult for the firms to find more complex products (since they always start in the periphery of the product space). However, they can also charge relatively higher prices due to their higher dispersion across the product space and the lower competitive pressure. From a theoretical viewpoint, this result is particularly interesting since many models implicitly assume a complete product space when they assume that any kind of innovation is possible at any time. The results here, however, indicate that studying deviations from this implicit special case are worth exploring since the other topologies differ not only with regard to the overall result, but are also associated with less inter-run variation.

For networks where the most complex products are in the centre of separated communities, such as the Barabási-Albert or the PLC network, it is easier for firms to find complex products, yet since this also comes with a stronger concentration of firms producing them, prices vary considerably. That is, firms tend to concentrate (or get stuck) in closely connected areas, which are more difficult to be left in favour of a different community of products – which might prevent them from diversifying into more lucrative production options.

The regular network takes an intermediate position, which is intuitive since there are no clusters in which firms can get stuck: products can be reached from everywhere, resulting in relatively high prices, less concentration but, therefore, also smaller average complexity. That is, while the regular network structure makes it – relative to the clustered Barabási-Albert or the PLC network – harder to identify complex products, it facilitates the evasion from competition, ultimately leading to higher prices.

The effects are quite the opposite on the ring network where firms concentrate on few products. Noticeably, they yield relatively lower prices and complexity values. This is due to the small neighbourhood structures: firms find it more difficult to move effectively towards more complex products, and get stuck more easily. Results are similar in the ‘Erdös-Rényi’ network: firms also show high levels of concentration, lower prices and higher complexity, yet, in all, inter-run variation is naturally very high for this kind of random network.

Taken all together, the results indicate that the topology of the product space, which can be thought of as a representation of the relatedness-structure of the products in the economy, does have an important impact on the innovation dynamics in the model. Complete networks, which are often implicitly assumed in models that model innovation as purely stochastic processes, imply rather distinct effects, therefore making an exploration of alternative topologies worthwhile. At the same time, the results suggest that while moving away from the complete network as such has considerable implications, the choice of the alternative complex topology is less crucial since the results for the alternative topologies are relatively more similar to each other.^15^

### Relevance of the allocation of complexity

**Fig. 4:**
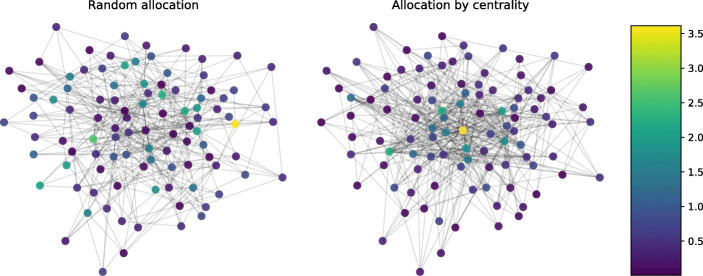
The left plot shows a product space with randomly allocated complexity values. On the right, more central products tend to have higher complexity

In real-world product spaces, more central products tend to be more complex (Hidalgo et al. 2007; Hidalgo and Hausmann 2009). Is this feature theoretically relevant for the dynamics of product innovation? In other words, does it matter whether product complexity is distributed randomly across the product space, as in the left network in Fig. 4, or whether complexity correlates with centrality such that the most central products are assigned the highest complexity values, as is the case in the right network in Fig. 4?^16^

**Fig. 5:**
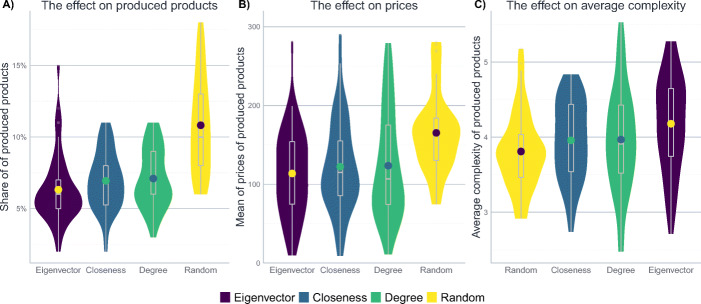
The effect of different allocation of product complexity. The figure shows median results after 500 steps of 50 iterations of the model, i.e. the situation during the final time step in Fig. 2. The other parameters are set to their baseline level described in Table 6. The topology of the product space is a Barabási-Albert graph. The width of the violins represents the rotated kernel density of the results, the point within the violin corresponds to its mean. Boxplots represent the median and the inter-quartile range

A nuanced answer is provided in Fig. 5: while it does not seem to matter much, according to which centrality measure the complexity values are allocated across the product space, there is a considerable difference between cases where complexity *does* correlate with some kind of centrality measure and where it does not (i.e. where complexity values are assigned randomly). If complexity values are allocated randomly, diversification is distinctly higher – firms do not tend to concentrate as much since paths on the network are less bottom-up, leading to scenarios where it becomes hard for firms to orient themselves on the product space. This results in a larger share of produced products and slightly lower levels of overall complexity. The latter does not translate into lower prices, however, since the minimal effect of lower complexity gets offset by the lower overall competition due to better diversification of firms. If, on the other hand, complexity does correlate with centrality, concentration as well as complexity levels are higher, indicating that it is easier for firms to identify and move to more complex products. This, however, also comes with a higher level of competition and, thereby, lower prices.

### The joint impact of topology and allocation

**Fig. 6:**
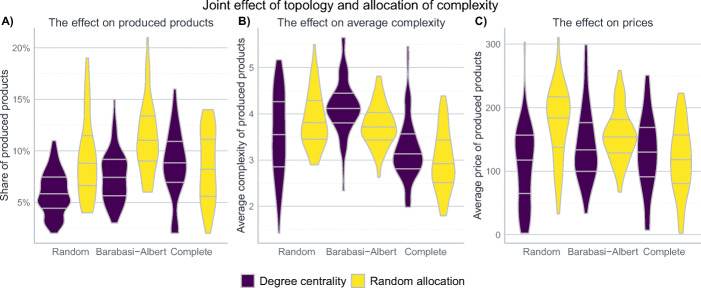
The joint effect of different topologies and allocations of product complexity. The figure shows the distribution of results after 500 steps of 50 iterations of the model for different topologies and different types of complexity allocations. The other parameters are set to their baseline level described in Table 6. The width of the violins represents the rotated kernel density of the results, the black lines the first, second, and third quartile

The previous two sections discussed the impact of the topology and the allocation of complexity values in a *ceteris paribus* manner. However, the impact of different allocation measures could be different, depending on the topology of the product space. To account for this fact, this section presents results for simulations in which both dimensions are changed simultaneously. The results are represented in Fig. 6. Since the results discussed in Section 4.2 indicate that the kind of centrality measure according to which complexity gets allocated is not essential, the random allocation of complexity values gets contrasted only with the allocation according to the degree centrality.

The first observation is that whether complexity gets allocated randomly or according to the degree centrality has no impact on the complete network. This is not surprising since the degree of every node is the same in this setting, making the allocation of complexity values effectively random as well.

The second observation is that both for the Erdös-Rényi as well as the Barabási-Albert network, a random distribution of complexity values comes with a higher share of produced products, indicating that a random allocation makes it more difficult for firms to search for more complex products in a systematic way. The impact on the average complexity is, however, different for the two network structures: in the Barabási-Albert network, an allocation of the complexity values according to degree centrality increases the average complexity – indicating that it is easier for firms to identify high-complexity products. The lower share of produced products comes with a focus on high-complexity products. This is different in the Erdös-Rényi network, where the effect is merely a greater dispersion of produced products that does not come with greater average complexity. Thus, it seems to be the case that only the core-periphery structure of the Barabási-Albert network allows firms to better identify and reach the higher-complexity products.

Finally, the effect on prices resembles the different effects on average complexity: in the Erdös-Rényi network, prices are considerably higher in the case of the random distribution, due to decreased competitive pressure. In case of the Barabási-Albert network, there is almost no effect on prices since the decrease in competitive pressure for the case of random allocation is offset by lower average complexity.

### Further sensitivity analyses

Finally, the sensitivity of the results to changes in the saturation threshold *q*^*max*^, the parameter *α*, and the maximum range of vision that the firms can attain (Υ_*max*_) is assessed.

**Fig. 7:**
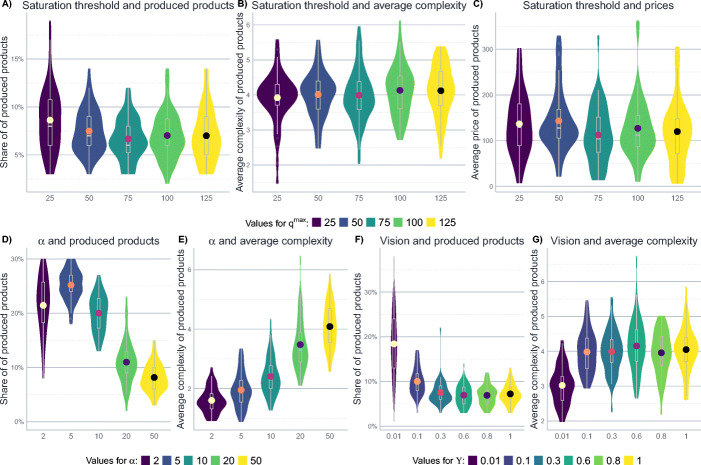
The effect of different values of *q*^*max*^, *α*, and Υ on the innovation dynamics. The figure shows median results after 50 steps of 50 iterations of the model. The other parameters are set to their baseline level described in Table 6. The topology of the product space is a Barabási-Albert graph. The width of the violins represents the rotated kernel density of the results, the point within the violin corresponds to its mean. Boxplots represent the median and the inter-quartile range

Panels A-C in Fig. 7 are concerned with the effect of the saturation threshold *q*^*max*^, and simply show that the model results are quite robust against changes in this parameter. The effect of *α* is more interesting: as indicated in Eq. 1, *α* controls the relevance of complexity for the prices of products. Panels D and E in Fig. 7 illustrate the effect of *α* on the share of produced products and average product complexity.

The first thing to note is a negative impact of *α* on the share of produced products. This is (1) due to the fact that a lower *α* translates into lower profits and lower returns on investment, leading to less investment-friendly scenarios, which is why firms get stuck more easily on products with low complexity, and, more importantly, (2) by the fact that complex products become relatively more attractive when complexity has a greater impact on prices. Thus, if *α* grows, firms cease to produce simple products and focus on the more complex ones, which is why the share of products produced decreases and average complexity increases.

Finally, panels F and G in Fig. 7 represent the implications of varying levels of the maximum share of vision the firms can attain (i.e. the maximum share of products that can be taken into account when navigating of the product space, Υ_*max*_). One can see that boundedly rational firms are doing quite well in exploring the product space: except for the cases with very limited sight, firms with a limited view on the product space do only slightly worse than firms that can attain a full picture of the product space, at least when we consider the share and the complexity of the produced products.

**Fig. 8:**
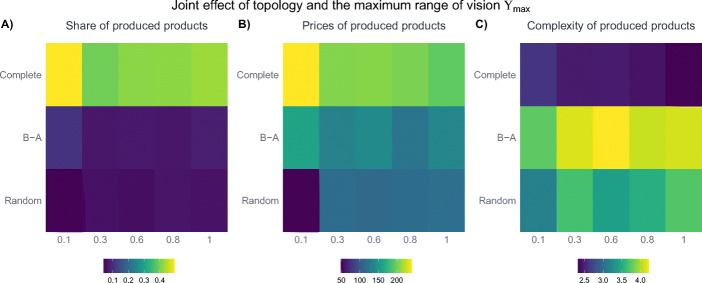
The joint effect of different topologies and the maximum range of vision Υ_*max*_. The figure shows median results after 500 steps of 50 iterations of the model, i.e. the situation during the final time step in Fig. 2. The other parameters are set to their baseline level described in Table 6

In order to examine whether the effect of Υ_*max*_ is different for distinct product space topologies, Fig. 8 distinguishes its impact on the complete, the Barabási-Albert, as well as the random network setting. The first thing to note is that the results are consistent with the ones of Fig. 7 in the sense that changes from very low to moderate levels of Υ_*max*_ are noteworthy, but that further increases have only negligible effects. Moreover, when it comes to the effect of different topologies, the results indicate that not only the overall share of produced products is highest in the complete network for the reasons explained above, but also the impact of variations in Υ_*max*_ are most pronounced for this kind of network: a low range of vision has especially severe consequences when the number of possible connections is large and the potential to get lost is high. For the other two networks, the effect is less pronounced but follows the same principle with the initial increase of Υ_*max*_ being most considerable.

## Discussion

Innovation and capability accumulation are important determinants of economic development. The empirical literature is very clear on this matter (e.g. Hidalgo et al. 2007; Hausmann et al. 2007; Dosi et al. 2019b). As has been shown in the concise literature review in Section 2, the determinants of capability accumulation are manifold. With regard to the more theoretical literature, we found that while various mechanisms have been formalized within microeconomic models, macroeconomic models largely consider innovation and capability accumulation processes in the context of *process innovation*, at least when it comes to the consumption good sector. Although the empirical literature has stressed its relevance, *product innovation* has received less attention so far in this context. Moreover, the relevance of product relatedness and the different kinds of capabilities have also not been integrated into evolutionary macroeconomic models so far, despite the fact that microeconomic models have already modelled a number of these mechanisms. One reason for this might be that microeconomic models considering product innovation in such a way are relatively complex and pay attention to “too many” mechanisms and processes to be translated into a macroeconomic context.

To advance the exchange between micro- and macroeconomic modelling, the goal in this paper was to come up with a simple supply-determined ABM, which captures some key ideas from the empirical literature on product innovation. This model might not only be expanded into a module for macroeconomic agent-based models, but was already used to to explore the theoretical implications of concepts such as the ‘principle of relatedness’ (Hidalgo et al. 2018). The focus has, so far, been on the topological structure of the product space, the relationship between product complexity and centrality, as well as the relevance of complexity for price determination. And although the model was kept as simple as possible, the results are interesting and show the relevance of these above-mentioned mechanisms for theoretical work.

In this context, the simplicity of the model is both an advantage and a drawback: the model design is obviously too simple to draw general conclusions, or to derive concrete policy implications. The absence of a household or government sector, a labour market, the simplistic price formation process and simplistic assumptions limit its applicability to real-world cases. Also, the lack of a labour market prevents the consideration of a number of essential elements of capability accumulation processes, such as learning-by-doing, individual learning, or the matching of firms to workers with the adequate specific skill set (see, e.g., Hötte 2021). Nevertheless, the model does suggest that when it comes to the design of innovation policies, tools that take into account the structure of technological spaces – such as the product space of Hidalgo et al. (2007) – are of particular value since the relatedness between products seems to be crucial, and neglecting it might lead to inaccurate assessments. It is unlikely that this result of the model vanishes once one takes into account additional elements such as the labour market or the government sector.

The immediate application to real-world cases is, however, not what the model was designed for in the first place: it was meant as an illustration of how a module for product innovation processes for macroeconomic ABM could look like. Extending the model by adding a household, a government sector and, most importantly, a labour market and then docking it into an existing macro ABM are logical next steps, but even in its current form the model has produced some interesting theoretical insights, which, however, also call for further investigation.

First, the structure of the product space as a measure for the relatedness of heterogeneous products has important implications and deserves further theoretical attention. The simulations show that even in a simple production economy in which the demand side is kept relatively primitive, the structure and distribution of product complexity *does* matter. Focusing exclusively on complete networks is insufficient if innovation dynamics are to be studied seriously within an evolutionary economic framework.

Second, the mechanisms affecting innovation dynamics are highly interdependent, even in the simplistic setting considered here. The relevance of product complexity for price formation, for instance, is closely interdependent with the possibilities of firms to evade competition, which, in turn, is dependent on the topology of the product space. Such results on the interdependency and context-dependency of central mechanisms is consistent with the empirical literature: innovation scholars regularly stress that innovation and capability accumulation are very context-dependent processes, which depend on many socio-cultural specificities and interact with numerous other socio-economic processes (see the summary in Aistleitner et al. 2021). For the present model, this means that although the results of the model and the provided interpretation in Section 4 are mostly intuitive, a more serious assessment requires the model to be embedded into a more comprehensive macroeconomic framework. At the same time, the simplicity of the model facilitates such undertaking. Moreover, the existing literature as well as the model above indicate that an agent-based approach provides a viable framework to address this challenge since it allows for the consideration of several distinct mechanisms and their interdependencies (see also Aistleitner et al. 2021). This optimistic interpretation is further strengthened by the possibility of approaching the challenge of modelling capability accumulation and product innovation in a modular manner: simple models, such as the present one, can be first developed and analyzed within a simplified environment and later, once they have been discussed and compared against alternatives, integrated into an existing macroeconomic modelling framework.

So, in all, while the simplicity of the current model prevents it from providing an acceptable account of how product innovation actually takes place, it can be a viable first step towards an adequate consideration of these processes within a more comprehensive macroeconomic framework.

## Conclusion

The paper introduced an ABM in which heterogeneous firms engage in various forms of capability accumulation and move on an artificial product space in the sense of Hidalgo et al. (2007). Since we were mainly interested in the processes underlying product innovation in the consumption good sector – a topic that so far has received less attention than process innovation, despite being highly relevant for economic development (e.g. Hausmann et al. 2007; Felipe et al. 2012) – the focus of the model was exclusively on the production side of the economy and did not include the consumption or the government sector. The model was used to investigate the impact of various topological structures of the product space on innovation dynamics. The results confirm our initial intuition that once product heterogeneity is introduced, and products are related to each other in non-trivial ways, innovation dynamics work very differently. While the empirical relevance of the ‘principle of relatedness’ in conjunction with product innovation processes has already been demonstrated (Hidalgo et al. 2007; Hausmann et al. 2007; Hidalgo et al. 2018), we hope to have stimulated the theoretical and model-based investigation of this subject.

That being said, the model implies some immediate avenues for future research, most of which relate directly to the second goal of our research, i.e. to bridge micro- and macroeconomic models of capability accumulation. An obvious next step is to use the present model as an innovation module within one of the existing agent-based macroeconomic models, as discussed in Section 5. This would not only allow for a more comprehensive analysis of the role of innovation for economic dynamics, but would also enable us to study how innovation dynamics interact with other relevant macroeconomic mechanisms. Second, by docking the model to an existing ABM, one can study how a model with homogeneous products and a focus on process innovation – such as most of the existing ABM – reacts to the consideration of product heterogeneity. Such an integration would also allow for a closer theoretical exploration of the mechanisms that link product innovation and economic development, a link of which the empirical literature has highlighted the importance but not illuminated the underlying mechanisms (e.g. Hidalgo et al. 2007; Felipe et al. 2012; Tacchella et al.^2013^).

An alternative course for future research is to remain within a more microeconomic context and to explore and extend the model along further dimensions: first, one could take the model more or less as it is and calibrate it directly with empirical data. For example, instead of using a theoretical product space, as is done in the present application, one could use an empirical one. Here, the number of products and the structure of their relatedness could be integrated into the model directly. Then, one could calibrate the model such that it replicates observed patterns of specializations over time. Such calibration exercise, however, would probably be more interesting after the model has been extended along the lines described above. Second, one could concentrate on the theoretical level and explore the interaction among firms and the mechanisms underlying knowledge spillovers in greater depth. One possibility is to allow for closer collaboration among firms, such as joint research and innovation projects. Another option would be to add workers to the model and investigate the effect of knowledge spillovers through worker migration (for empirical evidence see,e.g., Neffke and Henning 2013). Third, one could add the possibility of process innovation, such that firms not only can invest into the invention of new products, but also into the improvement of existing production processes. Having a model that features both different products as well as endogenous productivity dynamics would add much to the existing literature. Fourth, the consideration of different regions and inter-regional innovation dynamics has also been a prominent topic in the existing literature, albeit so far not in relation to a product space. Finally, and this concerns both a potential macro as well as micro variant of the model, the investigation of different sets of innovation policies is an obvious subject. This is particularly relevant if one is interested in products that differ not only in terms of their complexity but also in the amount of labour required or energy emitted – such a setting would be particularly interesting to explore nowadays, when environmentally friendly structural change is on the top priority list of policy makers.

In all, we hope that the model introduced here represents a small but first step in terms of both a closer integration of micro- and macroeconomic investigations of innovation and capability accumulation, as well as in advancing the modelling of innovation dynamics in the presence of heterogeneous products.

## Footnotes

1 In the capital good sector, both product and process innovation are regularly taken into account (see, e.g., Dosi et al. 2019a).

2 Arguably, the most comprehensive representation of an economy are the EURACE models of Dawid et al. (2019). But even here, only selected aspects of the overall economy are represented in greater detail.

3 For the effects of government activity, particularly public research, see, e.g., Dosi et al. (2018), for industrial policy, e.g., Dawid et al. (2018), and for the role of institutions, e.g., Caiani et al.(2019).

4 This is not product innovation in the narrow sense, but more in the spirit of the expanding variety models of Grossman and Helpman (1991b), where the focus is not on the invention of entirely new products but varieties of existing ones.

5 For a more detailed review see e.g. Aistleitner et al. (2021) or, for a review of macroeconomic ABM in general, Dawid and Delli Gatti (2018) or Dosi and Roventini (2019).

6 The distinction between new products and new product varieties, although intuitive at a first glimpse, is not easy to pin down. While delineating a general definition for the phenomena is beyond the scope of this paper, for the problem at hand it is sufficient to define a new product as one that is sufficiently different from other products both with regard to its complexity and the capabilities that are required to produce it.

7 In the beginning of the simulation, firms are allocated on the locations that have the smallest Eigenvector centrality. This ensures that firms start in the periphery of the product space, if such a periphery exists, and that they start on the simplest products possible, avoiding firms with little capabilities starting at products with very high complexity.

8 The results carry a broader theoretical relevance: many studies implicitly assume a complete product space with uniform distribution of complexity since all products are in principle the same and can be invented irrespective of the currently produced products. The results here help to illustrate the relevance of this common implicit assumption, which often turns out to be a peculiar special case.

9 Aside from the sections explicitly dedicated to study the implications of different topologies, the baseline topology used in this paper is that of a Barabási-Albert network since it is relative well-known and easy to understand and, additionally, captures the central empirical property of real-world product spaces, namely that of a core-periphery structure.

10 In the present model, complexity values are randomly drawn from a normal distribution. The model can easily implement other distributions of complexity values, yet we did not include this into the paper since no interesting implications for different distributions were found.

11 The common price setting strategies in macroeconomic ABM are summarized, e.g., in Dawid and Delli Gatti (2018).

12 This assumption is made to account for the fact that larger firms usually find themselves in more advantageous positions, making it easier for them to accumulate capabilities, see e.g. Aistleitner et al. (2021).

13 In this model, following the literature outlined in Section 2.4, the measures that firms can choose in order to enhance their capabilities are investment into *absorptive capacities* and into *R&D*. They will be explained below.

14 The repository can be reached via https://github.com/graebnerc/capability-accumulation-abm.

15 One might suspect that this comes from the relatively small product space with 100 products, but the results do not change qualitatively if one considers 500 products.

16 To construct product spaces in which complexity correlates with a centrality measure, the nodes are sorted according to a centrality criterion and the random complexity values are sorted according to their value. Then, the most central node is assigned the highest complexity value and so on.
